# Influence of Ag and/or Sr Dopants on the Mechanical Properties and In Vitro Degradation of β-Tricalcium Phosphate-Based Ceramics

**DOI:** 10.3390/ma16176025

**Published:** 2023-09-01

**Authors:** Junjian Che, Tao Sun, Xueman Lv, Yunhai Ma, Guoqin Liu, Lekai Li, Shengwang Yuan, Xueying Fan

**Affiliations:** 1 The College of Biological and Agricultural Engineering, Jilin University (Nanling Campus), 5988 Renmin Street, Changchun 130022, China; 2 The Key Laboratory of Bionic Engineering (Ministry of Education, China), Jilin University (Nanling Campus), 5988 Renmin Street, Changchun 130022, China; 3 Institute of Structured and Architected Materials, Liaoning Academy of Materials, Shenyang 110167, China; 4 Department of Ophthalmology, China-Japan Union Hospital of Jilin University, Changchun 130031, China; 5 Weihai Institute for Bionics, Jilin University, Weihai 264200, China

**Keywords:** β-tricalcium phosphate, sol–gel synthesis, bionic bone, compressive strength, in vitro degradation

## Abstract

β-tricalcium phosphate has good biodegradability and biocompatibility; it is widely perceived as a good material for treating bone deficiency. In this research, different contents of strontium (Sr) and silver (Ag) ion-doped β-tricalcium phosphate powders were prepared using the sol–gel method. After obtaining the best ratio of pore-forming agent and binder, the as-synthesized powders were sintered in a muffle for 5 h at 1000 °C to obtain the samples. Then, these samples were degraded in vitro in simulated body fluids. The samples were tested using a series of characterization methods before and after degradation. Results showed that the amount of Sr and/or Ag doping had an effect on the crystallinity and structural parameters of the samples. After degradation, though the compressive strength of these samples decreased overall, the compressive strength of the undoped samples was higher than that of the doped samples. Notably, apatite-like materials were observed on the surface of the samples. All the results indicate that Sr and/or Ag β-TCP has good osteogenesis and proper mechanical properties; it will be applied as a prospective biomaterial in the area of bone repair.

## 1. Introduction

With an aging population, changes in eating habits, diabetes and other health problems on the rise worldwide have caused great damage to our bones, leading to an increased incidence of bone damage or amputation [1,2,3]. As a result, many methods, including allografts, tissue engineering (TE) methods, and bioinert and bioactive implant materials, have been implemented to help patients regain limb use. However, the supply of allografts is limited and there is also the possibility of spreading disease and inflammation with their use. Meanwhile, remineralizing bone using TE methods is very time consuming. Bioinert implants made of cement, ceramic or metal are widely used in clinical practice as excellent alternatives. However, due to the mismatch between mechanical properties and stress, these implants easily fail after 10–15 years [4]. Bioactive implants, by contrast, can stimulate appropriate biological responses in the body. In particular, calcium phosphate implant materials are ideal bone conduction materials, but their mechanical properties are generally undesired [5]. Among them, calcium phosphate (CaP) compounds have high biocompatibility in the body and are very similar to the minerals that exist in bone and teeth; they are widely used in the field of health. For all these reasons, CaP compounds are generally regarded as the most attractive compounds for bioceramics [6]. The most prominent materials among the CaPs are hydroxyapatite (HA, Ca_10_(PO_4_)_6_(OH)^2^) and tricalcium phosphate (TCP, Ca_3_(PO_4_)_2_) [7,8,9], which are chemically similar to the minerals existing in bone. Similarly, the components of these two calcium phosphates are close to those of natural bones, so cells easily adhere to and proliferate on their surfaces. HA, as a metal implant medical coating material, has been used for more than 40 years. However, the clinical application of HA synthesis is limited by poor mechanical properties, poor chemical stability and poor thermal stability in acidic environments [10]. By contrast, β-tricalcium phosphate (β-TCP, Ca_3_(PO4)_2_) has high bioactivity and excellent osteoconductivity, and is a promising candidate for bone repair [10,11,12,13]. Numerous evaluations have shown that β-TCP imposes a positive effect on cell attachment, differentiation and proliferation [14,15,16,17]. However, shortcomings such as low compressive strength [18] and technological difficulties of preparation hinder its further application in loadbearing bone reconstruction [19].

It is very crucial to add trace elements to bioceramics. Magnesium (Mg), zinc (Zn), aluminum (Al), iron (Fe) and strontium (Sr) exist in the form of trace elements in the human body and contribute to accelerating the rate of bone formation. By this means, the structural characteristics of bioceramics can be improved or controlled by adding these elements to stimulate bone repair [20,21,22,23,24,25,26,27,28,29]. For example, doping bioceramics with specific metal ions could enhance the strength and degradation of β-TCP [30,31,32,33,34,35,36].

As an essential trace element, 99% of the Sr in the human body exists in bone, which is beneficial for balancing bone formation and bone resorption in bone metabolism [37]. Sr can partially replace Ca^2+^ sites in the crystal structure of β-TCP and thus change its biological, physical and chemical properties [38]. Meanwhile, Sr and Ca share the same physiological pathway, indicating that Sr can be deposited into the mineral structure of bone, especially in the high-metabolic-turnover region [39]. Furthermore, Sr is capable of promoting the apoptosis of osteoclasts, proliferation of osteoblasts and collagen synthesis [40]. Therefore, bioactive implants doped with strontium hydroxyapatite have been investigated for their use in bone repair [41,42,43]. Guo et al. [44] reported that the mechanical strength of tricalcium phosphate doped with Sr was significantly improved. Hu et al. [39] found that Sr-containing HA scaffolds had good biological activity, which can improve the proliferation rate of MG-63 osteoblast cells. Moreover, it can promote the differentiation of rat bone marrow mesenchymal stem cells (BMSCs9).

Although silver (Ag) ions and compounds have been shown to be toxic to certain bacteria, viruses, algae and fungi, silver, another essential trace element, is almost completely harmless to humans. Silver-substituted tricalcium phosphate (Ag-TCP) has been researched for a long time [45,46]. Song et al. [47] reported that all the Ag-TCP films in their study exhibited good antibacterial activity independently of Ag and no cytotoxicity was detected at the lowest concentration of the Ag-TCP coating. Roy et al. [48] found that the highest Ag concentration (0.5 M) had the best bacteriostatic effect but was cytotoxic. However, the silver concentration of 0.1 M had good antibacterial properties. Turkoz et al. [49] reported that Ag^+^ and F^−^ ion co-doped HA had the highest microhardness (0.5Ag1F). The HA contained a large number of Ag+ ions and showed antibacterial properties against Escherichia coli (*E. coli*).

However, most of the studies on Sr- and/or Ag-doped calcium phosphate materials focus on Sr- and/or Ag-doped hydrogenation, and there are few studies on Sr- and/or Ag-doped TCP. In addition, the degradation characteristics of Sr and/or Ag are neglected. In this paper, Sr-Ag-TCP with different contents was prepared using the sol–gel synthesis method. The changes in the mechanical properties and in vitro degradation of synthetic materials after Sr and/or Ag substitution were also studied and the related mechanical properties were discussed. Herein, the suitable strontium and silver carriers had an important influence on the biodegradability and generalization ability of β-TCP bone cement, which provides a reference for improving the biodegradability of β-TCP.

## 2. Materials and Methods

### 2.1. Powder Synthesis

In this research, calcium nitrate tetrahydrate (Ca(NO_3_)_2_·4H_2_O, Sinopharm, Shanghai, China), 2-phosphonobutane-1,2,4-tricarboxylic acid (PBTC, C_7_H_11_O_9_P, Sinopharm, Shanghai, China), strontium nitrate (Sr(NO_3_)_2,_ Sinopharm, Shanghai, China) and silver nitrate (AgNO_3,_ Sinopharm, Shanghai, China) were used as sources of Ca, P, Sr and Ag, respectively. For each sample, the molar ratio of (Ca+Sr+Ag)/P was adjusted to 1.50. The synthesized sample was named xSr-yAg-TCP, where x and y represented the molar ratios of Sr and Ag, respectively. The synthetic samples were referred to as: TCP, 1Sr-TCP, 1Sr-0.8Ag-TCP and 1Sr-3Ag-TCP. Firstly, calcium nitrate solution, strontium nitrate and silver nitrate powder were slowly added into PBTC solution. Anhydrous ethanol (C_2_H_5_OH, Sinopharm, Shanghai, China) was used as the solvent for each solution. The as-prepared mixture was vigorously stirred in a magnetic stirrer for 3 h. The temperature of the magnetic stirrer was controlled at 90 degrees Celsius. To produce precursor powders, the synthesized gel precursor was dried in a vacuum oven for 12 h. Finally, the obtained dry powders were sintered in a muffle furnace (NHK-170, Nittokagaku, Japan) at 1000 °C for 5 h. Elemental analysis of the synthesized products was performed by means of inductively coupled plasma optical emission spectrometry (ICP-OES) using an ICP-OES spectrometer (ICP-OES5110, Agilent, Santa Clara, CA, USA). 

### 2.2. Synthesis of Bone-like Porous Ceramics

The as-obtained powders were mixed with carbon powders (C, Sinopharm, Shanghai, China) and polyvinyl alcohol (PVA, [C_2_H_4_O]_n_, Sinopharm, Shanghai, China) in different quantities. The mixture was placed into a homemade mold and vibrated gently to compact it. After that, the powders were uniaxially compressed into a cylinder with a diameter of 10 mm. Then, the as-obtained samples were sintered in a muffle furnace to form the Sr- and/or Ag-doped β-TCP bone-like porous materials. The heating rates within the ranges 0 °C~200 °C, 200 °C~250 °C, 250 °C~400 °C and 400 °C~1000 °C were 3 °C/min, 2 °C/min, 3.5 °C/min and 3 °C/min. Their corresponding maintenance times were 40 min, 25 min, 40 min and 300 min, respectively. Finally, the samples were naturally cooled to room temperature.

### 2.3. Specimen Characterization

An X-ray diffractometer (XRD-6100, Shimadzu, Japan) was used for phase analysis of the sintered samples at 30 kV and 20 mA. Data were collected for 2θ ranging between 10° and 70° under CuKα radiation (λ = 1.5418 Å). The step size was 0.01° and the speed was set as 1 °/min. The crystallinity of the sintered powders was calculated according to the description elsewhere [50]. The FT-IR spectrum of the powders (FTIR-8400S, Shimadzu, Japan) was recorded in the 400–4000 cm^−1^ region. The resolution of the laser for collecting FT-IR spectra was 0.1~0.5 cm^−1^. A laser particle size distribution analyzer (BT-9300ST, Bettersize, Dandong, China) was used to determine the particle size of the synthetic powders. The micromorphology of the samples was determined using scanning electron microscopy (EVO18, Carl Zeiss, Jena, Germany). The test standard of the compressive strength of the ceramic samples was GB/T 4740-1999, China. The samples were cut into cylinders with a diameter of 5 mm and a height of 10 mm and their mechanical strengths were obtained using a mechanical testing machine (E43.104, RTEC, San José, CA, USA). The test was conducted at room temperature and the loading speed of the beam was set at 1 mm/min until the sample was broken. Each sample was subjected to 10 repeated tests, and the average value was taken as the test result.

### 2.4. In Vitro Degradation 

The sample was formed into a disk with a diameter of 10 mm and a height of 2 mm for the in vitro degradation test. In this study, the degradation performance of the Sr-Ag-TCP porous material was tested in 1.5 times simulated body fluid (SBF) solution according to Kokubo and Takadama [51]. Specifically, the SBF solution was a supersaturated solution of apatite containing NaCl, NaHCO_3_, KCl, K_2_HPO_4_·3H_2_O, MgCl_2_·6H_2_O, CaCl_2_ and Na_2_SO_4_. First, the sample was placed in a deionized water environment and impacted with ultrasonication. Then, the sample was soaked in 75% alcohol to ensure that there were no impurities in the pores. After that, the obtained sample was dried in a vacuum oven. Then, the sample was weighed on a balance (AL-204, METTLER TOLEDO, Shanghai, China) and put into a polypropylene plastic bottle. Subsequently, the 1.5SBF solution was added according to the ratio of the mass of porous biomimetic bone material 1 g to 100 mL solution. After sealing, the mixture was placed into a temperature incubator for the 28 d degradation test. To ensure the stability of the concentration of various ions in the 1.5SBF solution during the degradation test, the 1.5SBF solution in the bottle was replaced every 7 days throughout the test. During the degradation process, a balance was used to measure the quality of the sample. The pH of the degradation solution was measured using a pH meter (PHS-3C, INESA, Shanghai, China). 

## 3. Results and Discussion

### 3.1. Specimen Characterization

**Figure 1:**
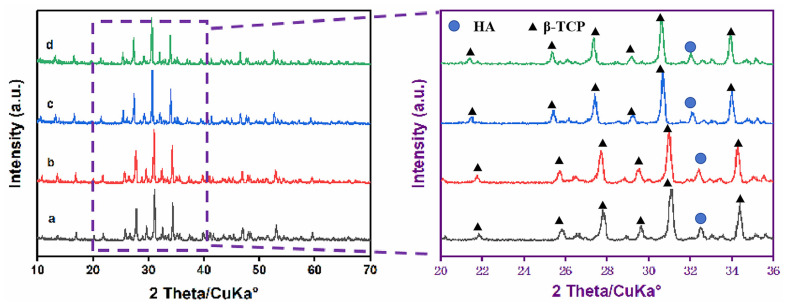
XRD patterns of the as-prepared β-TCP powders with different contents. (**a**) TCP, (**b**) 1Sr-TCP, (**c**) 1Sr-0.8Ag-TCP, (**d**) 1Sr-3Ag-TCP.

Figure 1 shows the XRD patterns of pristine β-TCP powders and the Sr^2+^- and/or Ag^+^-modified β-TCP powders prepared in this experiment. For all samples, we were able to observe the characteristic peaks of β-TCP (JCPDS PDF No: 09–0169) at 26.8°, 32°and 34° [41]. The major phase of β-TCP and the minor one of HA (JCPDS PDF No: 09–0432) were detected for all samples [52]. The phase composition was affected by the addition and amounts of both dopants. With the introduction of these metal ions, the characteristic peaks of the β-TCP powders shifted slightly to low angles, and the degrees of crystallization were good. This indicated that β-TCP powders with good crystallinity and Sr^2+^- and/or Ag^+^-modified β-TCP powders can be successfully prepared using the method adopted in this study. With the increase in the introduced amount of Ag^+^, the characteristic peak gradually shifted to a lower angle, and the intensity exhibited a minor change as well. The diameters of Sr^2+^, Ag^+^ and Ca^2+^ were 0.118 nm, 0.115 nm and 0.099 nm, respectively [42,53,54]. In the process of introducing Sr^2+^ and Ag^+^ into β-TCP, Sr^2+^ and Ag^+^ replaced the Ca^2+^ of β-TCP, leading to the linear expansion of the β-TCP lattice constant. Therefore, the characteristic peaks gradually shifted to a lower angle and the spacing between crystal faces increased. 

**Figure 2:**
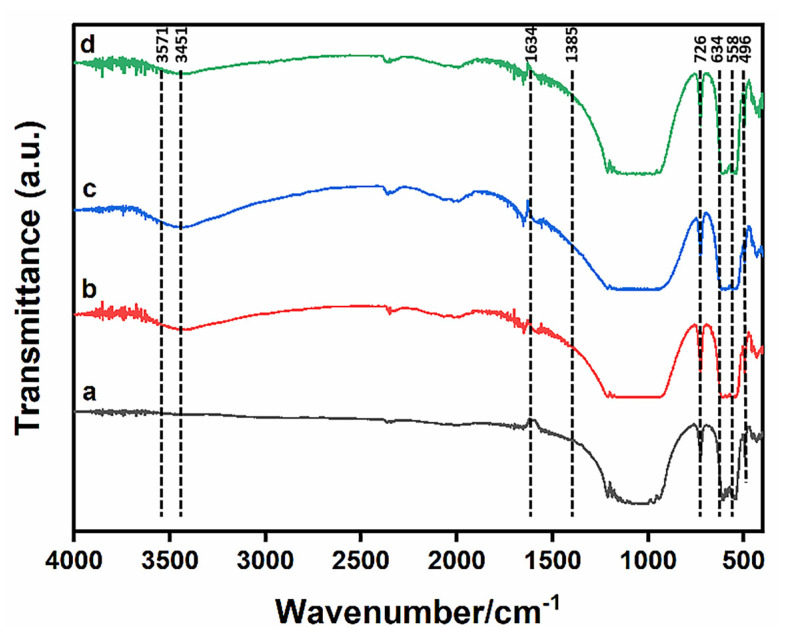
FTIR spectra of the as-prepared β-TCP powders with different contents. (**a**) TCP, (**b**) 1Sr-TCP, (**c**) 1Sr-0.8Ag-TCP, (**d**) 1Sr-3Ag-TCP.

Figure 2 displays the infrared spectra of Sr-doped and/or Ag-doped β-TCP powders. The resolution of the laser for collecting FT-IR spectra was 0.1~0.5 cm^−1^. The assignments of the observed bands on the FT-IR spectra were as follows: The bands at 496, 558, 613, 726 cm^−1^ and other ones within the spectral range of 900–1300 cm^−1^ were related to the vibrational modes of the phosphate groups [55]. The bands observed at 1634 and 3451 cm^−1^ were associated with the adsorbed water [56]. The band that stemmed from the carbonate group was detected at 1385 cm^−1^ [57]. The O-P-O bond bending vibration band was located at 500~650 cm^−1^ and the stretching vibration band of the P-O bond was situated at 940~1120 cm^−1^, which was consistent with β-TCP. It was verified that the β-TCP powders prepared via Sr^2+^ and/or Ag^+^ modification were mainly composed of β-TCP. For doped samples, the bands at 634 and 3571 cm^−1^, belonging to the characteristic vibrational modes of the hydroxyl groups for the HA phase, were detected. This was consistent with the detection of the HA phase formation in the XRD results [58]. The atomic radii of Sr^2+^ and Ag^+^ were larger than that of Ca^2+^. As Sr^2+^ and Ag^+^ entered the β-TCP lattice, the symmetry of the original lattice structure was affected, which contributed to a reduction in the absorption band intensity and vibration frequency of functional groups. 

**Figure 3:**
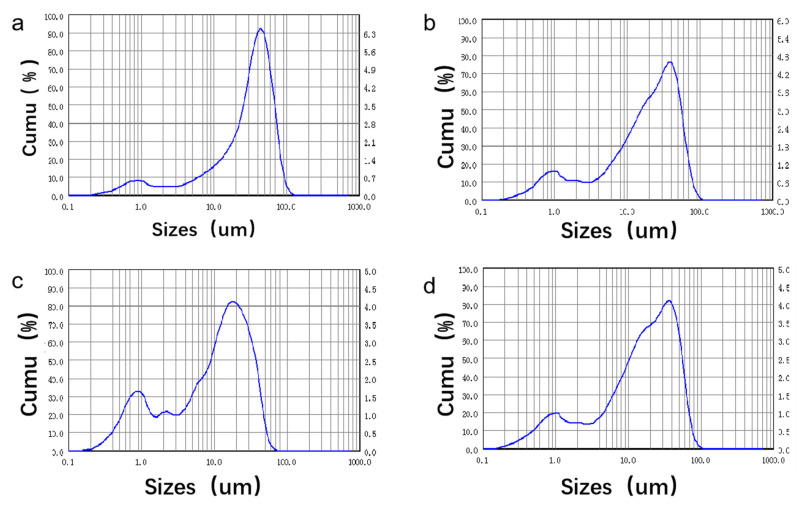
Particle size distribution of Sr-Ag-TCP particles with different contents. (**a**) TCP, (**b**) 1Sr-TCP, (**c**) 1Sr-0.8Ag-TCP, (**d**) 1Sr-3Ag-TCP.

It can be seen in Figure 3 that the pure β-TCP powder with a particle size below 40 μm accounted for 91.34% of the total content. The 1Sr-β-TCP powder composed of particles less than 40 μm in size accounted for 94.77% and the 1Sr-0.8Ag-TCP powder with a particle size below 40 μm accounted for 92.35% of the total content. The 1Sr-3Ag-TCP powder composed of particle sizes less than 40 μm accounted for 91.76% of the total content. In brief, most of the as-prepared powders had a particle size of less than 40 μm, which met the preparation requirements for bioceramics.

In order to confirm the chemical composition of the synthesized compounds, elemental analysis was performed by means of ICP-OES. The results of the analysis are summarized in Table 1.

### 3.2. Compressive Strength 

**Figure 4:**
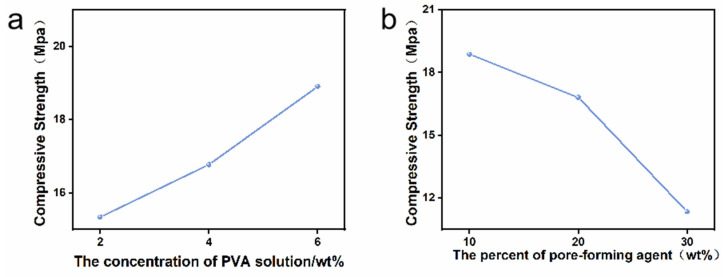
(**a**) Compressive strength of porous materials with 20 wt% pore-forming agent content at different PVA concentrations of 1Sr-TCP. (**b**) Compressive strength of porous materials with 6 wt% PVA concentration at different pore-forming agent contents of 1Sr-TCP.

Figure 4a exhibits the distribution of the compressive strength of 1Sr-TCP ceramic materials with different contents of pore-forming agent when the binder concentration was 6 wt%. With the increase in the pore-forming agent, the compressive strength of the porous ceramics decreased sharply. When the amount of carbon powder was 10 wt%, the compressive strength reached the highest value (18.87 MPa). The compressive strength decreased sharply to 11.34 MPa when the amount of carbon powder was 30 wt%. As the amount of pore-forming agent increased, the size and number of mesopores increased, which affected the mechanical properties and mechanical strength of the ceramic materials.

Figure 4b reveals the distribution of the compressive strength of 1Sr-TCP ceramic materials with different binder concentrations (2 wt%, 4 wt%, 6 wt%) when the amount of pore-forming agent was 20 wt%. The compressive strength of the ceramics was enhanced with the increasing PVA concentration. When the concentration of PVA increased from 2 wt% to 6 wt%, the compressive strength increased from 15.34 MPa to 18.91 MPa. Generally, the PVA solution with a low concentration led to uniform contact between the PVA and the as-prepared powders and thus a weak bonding strength, which eventually reduced the compressive strength of the ceramic materials. On the contrary, the PVA solution with a high concentration increased the interaction force between the powders, which ultimately endowed the ceramic materials with a high compressive strength. The optimum ratio of pore-forming agent to binder was determined using the single-factor test, with the content of pore-forming agent being 20 wt% and the concentration of PVA being 6 wt%.

**Figure 5:**
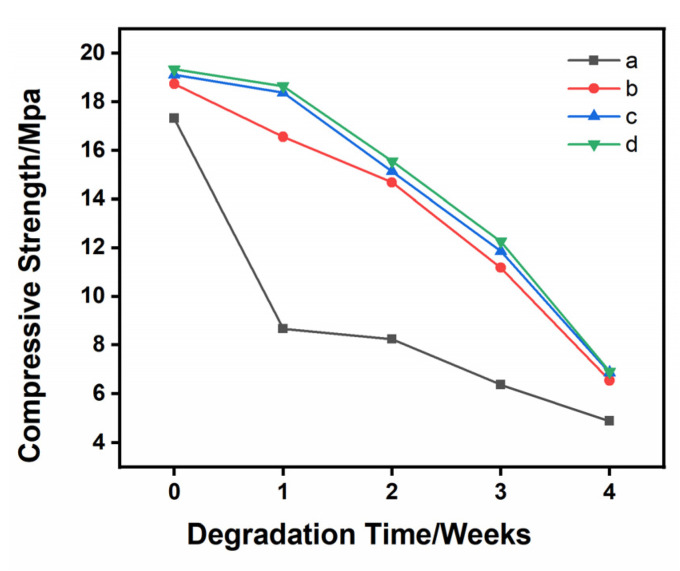
The compressive strength changes of four samples during the degradation process when the content of pore-forming agent is 20 wt% and the concentration of PVA is 6 wt%. (**a**) TCP, (**b**) 1Sr-TCP, (**c**) 1Sr-0.8Ag-TCP, (**d**) 1Sr-3Ag-TCP.

Figure 5 shows the variation in the compressive strength of the four specimens during the degradation process. Before degradation, 1Sr-3Ag-TCP had the highest compressive strength (19.34 MPa), while pure TCP had the lowest compressive strength (17.32 MPa). All samples showed a slow and steady degradation of compressive strength during degradation. After degradation, the compressive strength of 1Sr-3Ag-TCP was the highest (6.90 MPa), while that of pure TCP was the lowest (4.88 MPa). All the compressive strengths of the ceramic materials slightly decreased; the compressive strength of the 1Sr-3Ag-TCP ceramic material was always the highest during this process, while the compressive strength of the TCP was always the lowest. Overall, with the addition of Sr^2+^ and Ag^+^, the compressive strength of the material was always higher than that of undoped samples in the degradation process. And the higher the doping amount was, the higher the compressive strength became. This was because the addition of strontium ions and silver ions changed the structure of the original lattice, resulting in lattice distortion and thereby increasing the compressive strength of the ceramic material.

### 3.3. Specimen Characterization after Degradation

**Figure 6:**
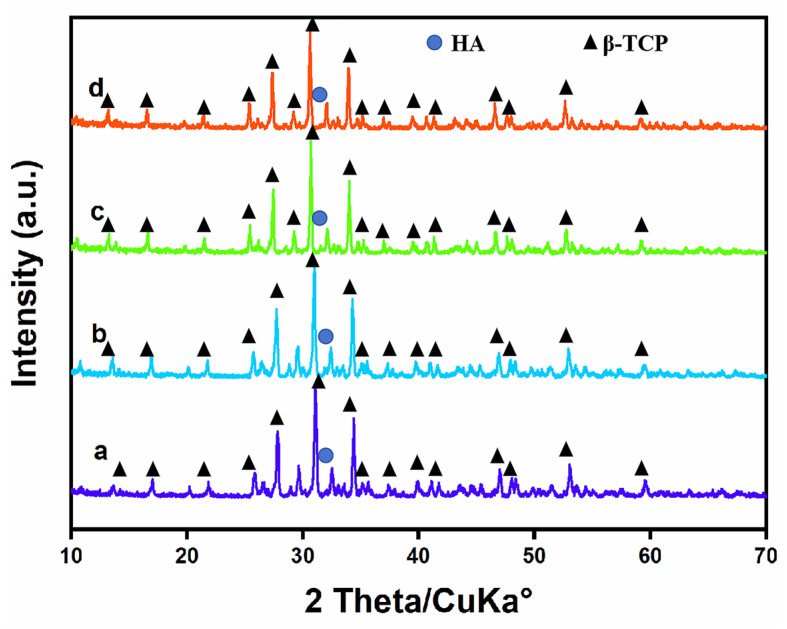
XRD patterns of the four samples after degradation when the content of pore-forming agent is 20 wt% and the concentration of PVA is 6 wt%. (**a**) TCP, (**b**) 1Sr-TCP, (**c**) 1Sr-0.8Ag-TCP, (**d**) 1Sr-3Ag-TCP.

Figure 6 exhibits the XRD testing results of Sr- and/or Ag-doped β-TCP samples after degradation in 1.5× SBF solution for 28 d. The crystal structure of the surface materials showed no obvious change after degradation in comparison with the materials without degradation. The XRD patterns of the degraded materials contained obvious β-TCP characteristic peaks, and the diffraction peaks were enhanced at 26.8°, 31–32.5°, 32° and 34°, which were similar to the apatite diffraction peaks. This indicated that apatite-like materials were formed on the materials during the degradation reaction. In addition, the XRD results demonstrated that the crystallinity of such materials increased with the increasing amounts of Sr and Ag. The doping of Sr and Ag played a vital role in the deposition ability of β-TCP-induced apatite-like materials. The formation of apatite-like materials indicated that Sr- and/or Ag-doped β-TCP ceramic materials had good osteoconduction and biocompatibility.

**Figure 7:**
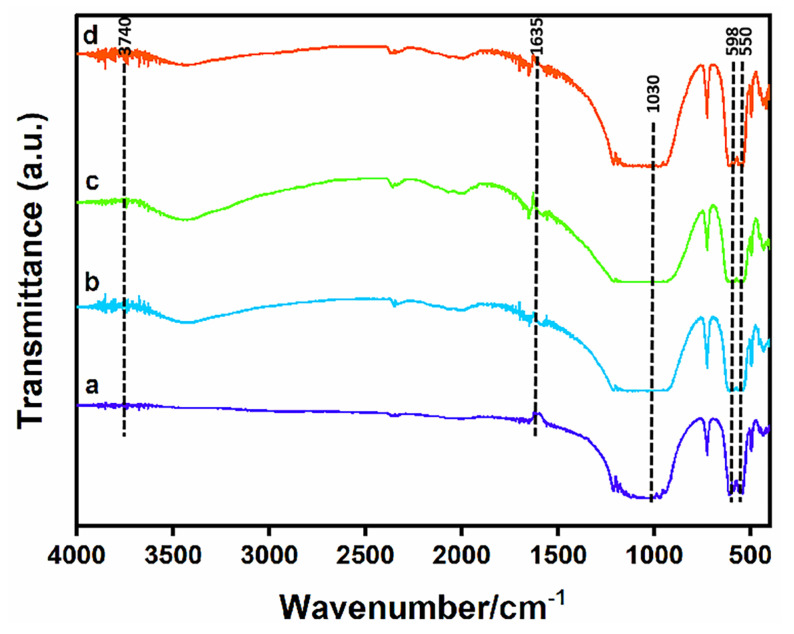
FTIR spectra of the four samples after degradation when the content of pore-forming agent is 20 wt% and the concentration of PVA is 6 wt%. (**a**) TCP, (**b**) 1Sr-TCP, (**c**) 1Sr-0.8Ag-TCP, (**d**) 1Sr-3Ag-TCP.

Figure 7 displays the FTIR spectra of the samples after degradation. It can be seen that H_2_O vibration bands appeared at 3740 cm^−1^ and 1635 cm^−1^, and phosphate ion (PO_4_^3−^) vibration bands appeared at 550 cm^−1^, 598 cm^−1^ and 1030 cm^−1^. A carbonate (CO_3_^2−^) vibration band appeared at 1633 cm^−1^. Compared with the samples before degradation, the intensity of the bands increased obviously, which confirmed that apatite-like materials were formed on Sr- and/or Ag-doped β-TCP ceramic materials. This also confirmed the conclusion from the XRD analysis that the bioactivity of the material was strongly enhanced.

### 3.4. Material Quality Increases after Degradation

**Figure 8:**
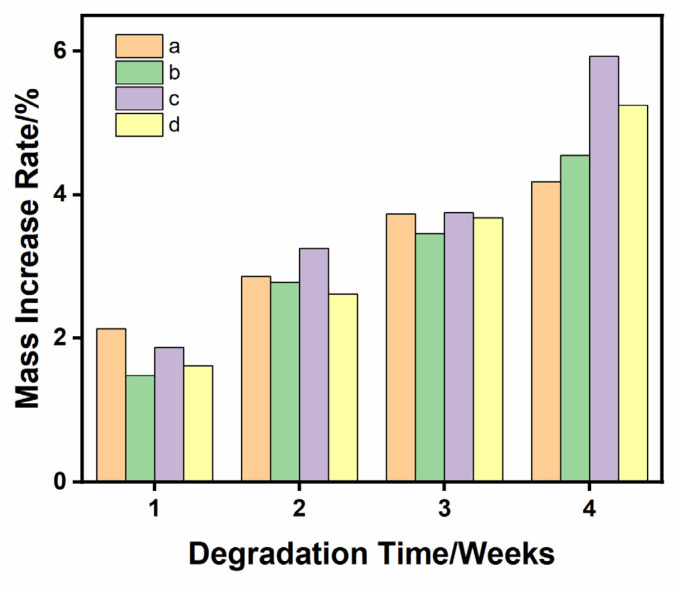
Mass changes of four samples during degradation when the content of pore-forming agent is 20 wt% and the concentration of PVA is 6 wt%. (**a**) TCP, (**b**) 1Sr-TCP, (**c**) 1Sr-0.8Ag-TCP, (**d**) 1Sr-3Ag-TCP.

The quality changes in the as-prepared samples in 1.5 times SBF solution for 1, 2, 3 and 4 weeks are shown in Figure 8. During the degradation process of 4 weeks, all the samples were in the state of weight gain, indicating that apatite-like materials were formed on their surfaces. This was mainly because of the synergistic effect between the mineral deposition process and the degradation process. During the degradation process, Ca^2+^ and PO_4_^3−^ were released from the β-TCP ceramic and re-engaged in the mineralization process. After 4 weeks, the weight gain rate of the 1Sr-0.8Ag-TCP ceramic was the highest at 5.93% ± 21% and the weight gain rate of the β-TCP ceramic was the lowest at 4.18% ± 0.11%. The weight gain rate of the 1Sr-3Ag-TCP ceramic was 5.25% ± 0.03%., and the weight gain rate of the 1Sr-TCP ceramic was 4.55% ± 0.17%. Due to the introduction of Sr^2+^ and Ag^+^, the sample weight gain rate increased. It was confirmed that the introduction of Sr^2+^ and Ag^+^ was beneficial for the growth of the mineralization and deposition of the apatite layer onto the β-TCP ceramic surface. Moreover, a small amount of silver contributed to a faster mineralization deposition process. 

**Figure 9:**
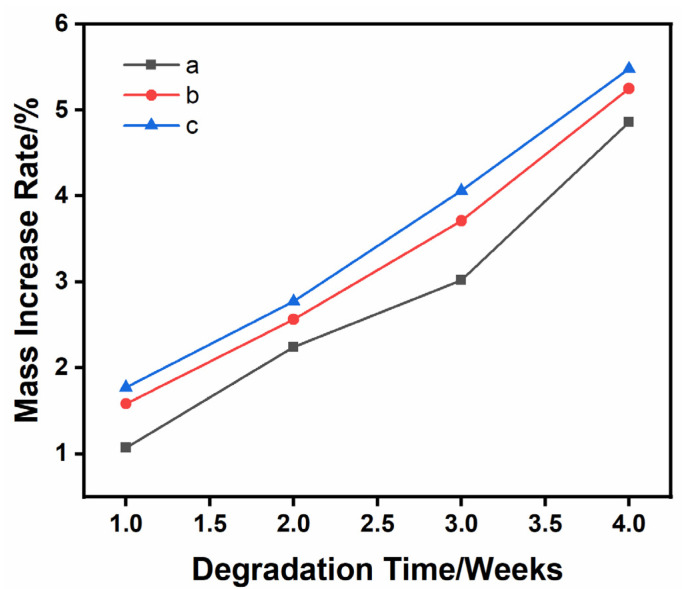
The quality change in 1Sr-3Ag-TCP with different pore-forming agent contents during degradation. (**a**) 10% pore-forming agent; (**b**) 20% pore-forming agent; (**c**) 30% pore-forming agent.

The quality changes in 1Sr-3Ag-TCP ceramic materials with different amounts of pore-forming agent immersed in 1.5 times SBF solution for 4 weeks are illustrated in Figure 9. It can be seen that the quality of all samples increased during the degradation process, and that, as the porosity increased, the rate of mass increase after degradation was significantly accelerated. The porosity of the samples affected the weight gain rate of the samples. The higher the porosity of the porous ceramic samples became, the more that the SBF solution permeated into the samples, meaning that the degradation occurred simultaneously on the surface and inside of the samples and that more ions were released. Then, they was rapidly deposited onto the surface to form apatite-like materials, which showed a higher rate of weight gain.

### 3.5. pH Changes during Degradation

**Figure 10:**
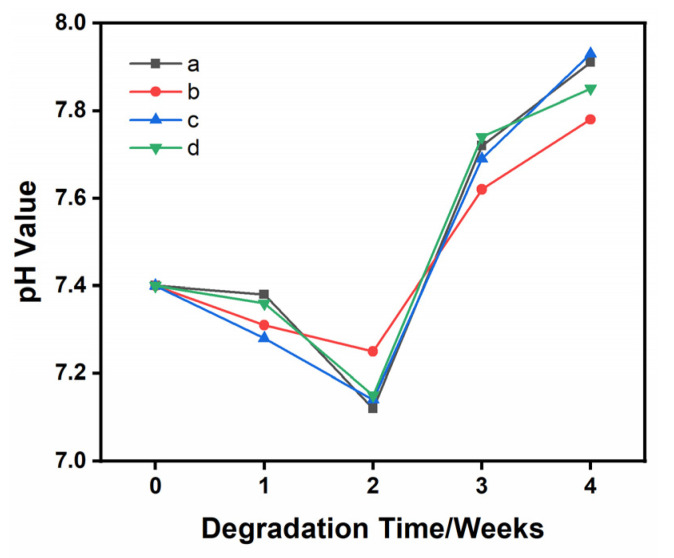
pH changes in the four samples during degradation. (**a**) TCP, (**b**) 1Sr-TCP, (**c**) 1Sr-0.8Ag-TCP, (**d**) 1Sr-3Ag-TCP.

The pH change curve of the SBF solution in the degradation process is shown in Figure 10. The pH values of the SBF solution with different immersed ceramic materials (TCP, 1Sr-TCP, 1Sr-0.8Agβ-TCP, 1Sr-3Ag-TCP) were similar during the degradation test for 28 days. It was confirmed that the excellent mineralization properties of β-TCP ceramic materials were not affected by the introduction of Sr^2+^ and Ag^+^. In the first 7 days, the pH value was between 7.3 and 7.5, and there was no significant change. In the second 7 days of this process, the pH value decreased to 7.0~7.1. This might be due to the degradation of ceramic materials under the action of SBF immersion and the generation of acidic degradables, measures which reduce pH value. In the third 7 days of this period, the pH value increased gradually, the ceramic material not only degraded under the action of SBF immersion but also formed an apatite-like material layer on the surface, and the deposition rate was faster than the degradation rate. Until the final week of this process, the pH value increased to 7.8~7.9, which was beneficial for accelerating the deposition rate of apatite-like materials in alkaline environments with higher pH values. The dissolution of Ca^2+^ and P^5+^ in the SBF solution resulted in ion exchange [59]. The process of pH change was strongly correlated with the concentration of Ca^2+^ and P^5+^ in the SBF solution and the rate of mineralization and deposition of Ca^2+^ and P^5+^ [60]. As the concentration of Ca^2+^ and P^5+^ increased during this process, the degradation products were alkaline and the pH of the degradation solution increased [61].

### 3.6. SEM Observations after Degradation

**Figure 11:**
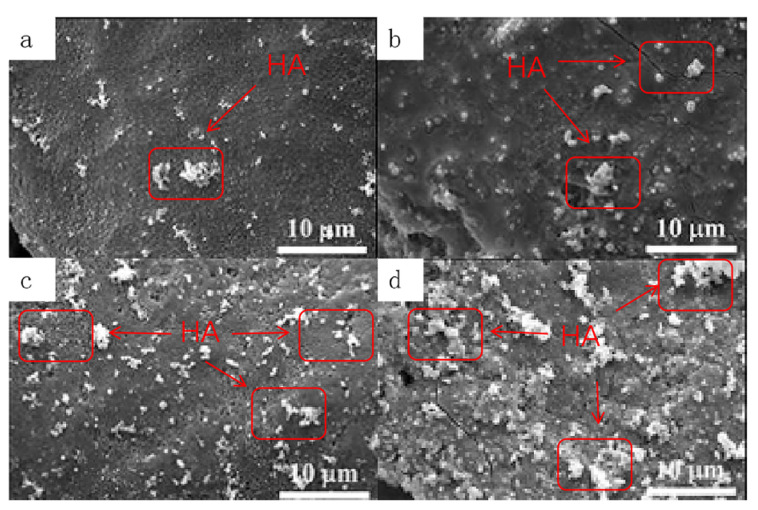
SEM images of four samples after degradation. (**a**) TCP, (**b**) 1Sr-TCP, (**c**) 1Sr-0.8Ag-TCP, (**d**) 1Sr-3Ag-TCP.

Figure 11 displays the SEM images of TCP, 1Sr-TCP, 1Sr-0.8Ag-TCP and 1Sr-3Ag-TCP after degradation. Cracks occurred in all samples, and these were caused by the degradation of the material. A certain amount of mineralization appeared on each sample and formed apatite-like materials, which had a smaller size and close packing, similar to hydroxyapatite. With the increase in Sr^2+^ and Ag^+^, the cracks on the surface of the sample increased and more apatite-like material on the surface could be observed. The apatite-like materials were partially agglomerated. Considering the XRD and FTIR results, the introduction of Sr^2+^ and Ag^+^ was conducive to the deposition of such materials onto the β-TCP ceramic surface.

## 4. Conclusions

In conclusion, Sr- and/or Ag-doped β-TCP powders without impurities were prepared via the sol–gel method. By conducting compressive strength tests, the optimum dosages of pore-forming and binder agents were ascertained. With increasing Sr and Ag contents, the compressive strength of the sample also increased. In vitro degradation experiments demonstrated that increasing the Sr and/or Ag doping contents was beneficial for the deposition of apatite-like materials. The quality of the deposited materials was obviously enhanced as the porosity increased when the content of Sr and/or Ag was constant. Therefore, the suitable strontium and silver carriers had an important influence on the biodegradability and mineralization ability of β-TCP bone cement. This study provides a reference for improving the biodegradability of β-TCP.

## Figures and Tables

**Table 1:** Results of the elemental analysis of the samples performed using ICP-OES.

Sample	Sr/(Sr + Ag + Ca), %	Ag/(Sr + Ag + Ca), %	(Sr + Ag + Ca)/P
TCP	-	-	1.51
1Sr-TCP	1.03	-	1.53
1Sr-0.8Ag-TCP	1.06	0.83	1.51
1Sr-3Ag-TCP	1.04	3.07	1.49

## Data Availability

We apologize for not being able to provide data due to privacy.
